# Much Craniotomy

**Published:** 1881-04

**Authors:** 


MUCH CRANIOTOMY.—" It is said the united obstetrical experience of Drs. Barker, Taylor and Thomas of New York; Penrose and Wallace of Philadelphia, and Chatard, Sr., of Baltimore, covers a period of two and a quarter centuries, during which they have attended at the births of over forty-five thousand children, and that they have been compelled to perform the operation of craniotomy less than twenty times. If these figures merely approximate correctness should not one who has not had twenty years experience, and who is said to have sacrificed 18 or more children by craniotomy, be reported to the "Society, for preventing cruelty to animals," for slaughtering the innocents?" Yours Fraternally, Q.

A. Such an one should be turned over to a Grand Jury rather.

